# Computer-Aided Discovery of Small Molecule Inhibitors of Thymocyte Selection-Associated High Mobility Group Box Protein (TOX) as Potential Therapeutics for Cutaneous T-Cell Lymphomas

**DOI:** 10.3390/molecules24193459

**Published:** 2019-09-24

**Authors:** Vibudh Agrawal, Mingwan Su, Yuanshen Huang, Michael Hsing, Artem Cherkasov, Youwen Zhou

**Affiliations:** 1 Vancouver Prostate Centre, Department of Urologic Sciences, Faculty of Medicine, University of British Columbia, 2660 Oak Street, Vancouver, BC V6H 3Z6, Canada; 2 The Bioinformatics Graduate Program, University of British Columbia, Vancouver, BC V5T 4S6, Canada; 3 Department of Dermatology and Skin Science, University of British Columbia, Vancouver, BC V5Z 4E8, Canada; 4 Dermatologic oncology program, BC Cancer, Vancouver, BC V5Z 1L3, Canada

**Keywords:** computer-aided drug design, cutaneous T cell lymphoma, TOX, HMG-box domain, small molecule inhibitors

## Abstract

Cutaneous T-cell lymphomas (CTCL) are the most common primary lymphomas of the skin. We have previously identified thymocyte selection-associated high mobility group (HMG) box protein (TOX) as a promising drug target in CTCL; however, there are currently no small molecules able to directly inhibit TOX. We aimed to address this unmet opportunity by developing anti-TOX therapeutics with the use of computer-aided drug discovery methods. The available NMR-resolved structure of the TOX protein was used to model its DNA-binding HMG-box domain. To investigate the druggability of the corresponding protein–DNA interface on TOX, we performed a pilot virtual screening of 200,000 small molecules using in silico docking and identified ‘hot spots’ for drug-binding on the HMG-box domain. We then performed a large-scale virtual screening of 7.6 million drug-like compounds that were available from the ZINC15 database. As a result, a total of 140 top candidate compounds were selected for subsequent in vitro validation. Of those, 18 small molecules have been characterized as selective TOX inhibitors.

## 1. Introduction

Cutaneous T cell lymphoma (CTCL) is a primary lymphoma of the skin that is derived from cutaneous resident memory T cells. In the vast majority of cases, the malignant T cells are CD4^+^ in nature. The most common variants of CTCL are mycosis fungoides (MF) and Sezary syndrome (SS). While most patients with early stage CTCL have a life span approaching that of healthy individuals, approximately 10% of CTCL patients with limited patch and plaque disease, and up to 25% of patients with extensive patches or plaques, eventually develop end-stage conditions such as leukemic stage CTCL including SS, which has high mortality. SS patients have a median survival of 2–4 years and an estimated 5-year survival rate of 24% [1]. The treatment of CTCL is individualized, depending on the disease severity and clinical stage. In early stages, CTCL mainly affects the skin; thus, the therapies available are skin-directed, including topical steroids, topical nitrogen mustard, and topical retinoids as well as phototherapy. In refractory cases, and in late stages when the disease is no longer limited to the skin, systemic therapies are often required. Although a number of agents, including retinoids, interferons (IFN), monoclonal antibodies, epigenetic modifiers such as histone deacetylase inhibitors (HDACi), and denileukin diftitox, have shown benefit in the treatment of advanced disease, none are curative. Therefore, new therapies for CTCL are urgently needed.

Thymocyte selection-associated high mobility group box protein (TOX) is a 526 aa nuclear protein (molecular weight, 57 kD) that binds DNA in a structure-dependent and sequence-independent manner [2]. It is a member of the evolutionarily conserved high mobility group (HMG) box family and a key regulatory nuclear protein in the development of CD4^+^ T cells, natural killer cells, and lymphoid tissue inducer cells [2,3,4,5,6]. The roles of TOX in immune system development are well-characterized [2]. Its expression is tightly controlled and expressed during early CD4^+^ T cell development. When mature CD4^+^ T cells exist the thymus and enter the peripheral sites, such as the blood and the skin, TOX expression is switched off [4]. Gene knockout experiments have demonstrated that mice lacking functional *TOX* gene are viable, and developmentally normal with the exception of their lacking CD4^+^ T cells, confirming the essential role of TOX in T cell development and maturation. In recent years, strong evidence has emerged that TOX is a specific biomarker, strong prognostic factor, key pathogenic driver, and attractive therapeutic target for CTCL [7,8,9,10].

(1) TOX is aberrantly expressed in CTCL: In comparative transcriptome studies [10] comparing CTCL skin biopsies with those of normal healthy skin and benign inflammatory dermatosis (BID), TOX emerged as the most highly enriched in CTCL skin biopsies. Similarly, purified CTCL cells expressed much higher levels of *TOX* mRNA compared with benign CD4^+^ T cells from healthy donors and from patients with BID. In addition, TOX-specific antibodies identified the malignant CD4^+^ T cells in the dermis and epidermis of CTCL, and in purified primary CTCL cells and in cultured cell lines. TOX is also highly expressed in Pautrier’s micro-abscesses, the pathological hallmark of CTCL [10]. (2) Enhanced transcript levels of TOX correlate with increased risk of disease-specific mortality in CTCL: Further experiments showed that TOX expression levels in CTCL skin biopsies and in peripheral-blood-purified malignant CTCL cells were positively correlated with disease-specific mortality of CTCL patients [9]. (3) Stable knockdown of *TOX* inhibits growth of CTCL cells in vitro: After *TOX* gene silencing using lentiviruses containing *TOX*-shRNA sequences, proliferation of CTCL cells (Hut78, HH, and SZ4), was markedly suppressed compared with control cells. Similarly, colony formation capability in vitro was decreased [8]. (4) TOX suppression induces apoptosis and caspase activation in CTCL cells: With TOX suppressed, CTCL cells had increased numbers of apoptotic cells. On the molecular level, *TOX* knockdown led to activation of caspase 9 and caspase 3, which are involved in apoptosis initiation and execution [8]. (5) TOX suppression impairs tumor-forming ability of CTCL cells in vivo: Using a well-established mouse model of CTCL developed in house, we found that CTCL patient-derived cell lines (Hut78 and HH) readily formed tumors when injected subcutaneously. However, when *TOX* expression was silenced, this ability was abolished [8], demonstrating the indispensable role of TOX in the pathogenesis of CTCL. (6) TOX suppression led to expression changes of multiple downstream genes: Transcriptomic analysis showed that *TOX* silencing resulted in marked expression changes of numerous genes, with the most significant changes observed in cell cycle suppressor gene *SMAD3*, which is normally suppressed by *TOX* but was sensitively induced after *TOX* gene silencing [8]. In summary, there are multiple lines of evidence that together strongly demonstrate that TOX is an attractive molecular target for developing CTCL therapies.

There are currently no small molecule inhibitors of TOX, and herein we aimed to address this unmet need by developing anti-TOX therapeutics through a computer-aided drug design (CADD) platform that we have previously established [11] and successfully utilized in a number of other cancer-related drug targets, including androgen receptor (AR) [12], estrogen receptor (ER) [13], ETS-related gene (ERG) [14], MYC [15], etc. Here, we report the use of this established CADD pipeline, which combined virtual screening of 7.6 million drug-like small molecules with in vitro experimental validation, to discover a new class of anti-TOX compounds.

## 2. Results

### 2.1. Druggability Assessment of the TOX HMG-Box Domain

**Figure 1:**
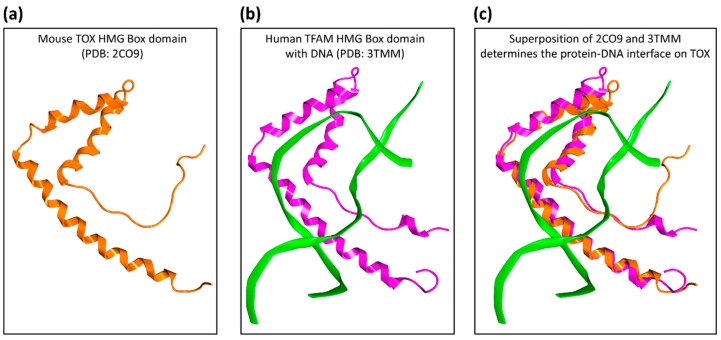
Protein structural templates for the high mobility group (HMG)-box domain of thymocyte selection-associated high mobility group box protein (TOX). (**a**) A NMR structure of mouse TOX protein (PDB ID: 2CO9) was identified as the best structural template, with 100% sequence similarity across the 87 amino acids of the HMG-box domain, compared to the human TOX protein. (**b**,**c**) By superimposing the 2CO9 structure (orange ribbons) onto the HMG-box protein Transcription factor A, mitochondrial (TFAM, pink ribbons, PDB ID: 3TMM, 46% sequence similarity to human TOX, 3.8A RMSD) in complex with DNA (green ribbons), the TOX–DNA interface was determined.

**Figure 2:**
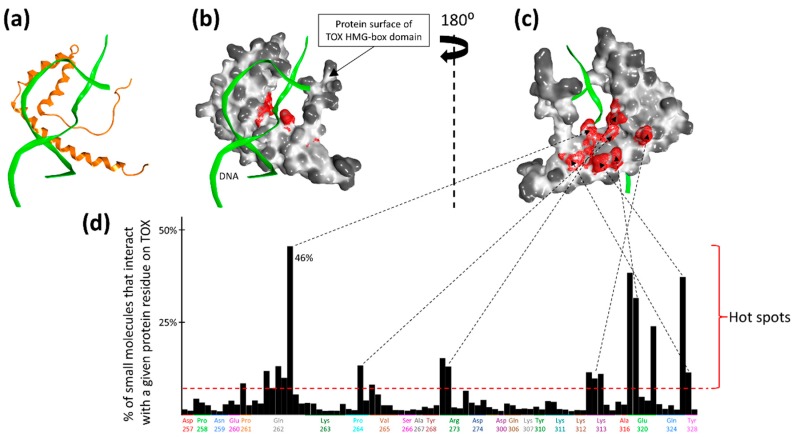
Druggability of the HMG-box domain of TOX. (**a**) TOX–DNA interface as determined from Figure 1C. TOX HMG-box domain in orange ribbons, DNA in green ribbons. (**b**) Molecular surface presentation of the TOX HMG-box domain, in the same orientation as (**a**). (**c**) The TOX HMG-box domain is rotated by 180° to illustrate the small-molecule binding hot spots (red). (**d**) A total of 200,000 drug-like molecules were docked to the TOX HMG-box domain. The percentage of interacting small molecules is shown for each protein residue as a bar graph (multiple interactions/contacts are represented as separate bars for each amino acid). Protein residues, including Gln262, Pro264, Arg273, Lys313, Glu320, Gln324, and Tyr 328, that interacted with at least 10% of the small molecules, have been highlighted and mapped to their corresponding locations as hot spots (red surface patches) on the TOX HMG-box domain.

The available NMR structure of TOX deposited in the PDB database (ID: 2CO9) [16], as shown in Figure 1, was used as a model for in silico screening of small molecules. To investigate the druggability of the DNA-binding HMG-box domain of TOX, we performed initial in silico screening of 200,000 drug-like chemical structures from the ZINC15 database [17] by using docking software Glide [18] with a ‘blind docking’ setup where no specific binding site was pre-defined. Figure 2 illustrates that the binding of these 200,000 virtual compounds concentrated on a few “hot spots” located at the DNA interface on the HMG-box domain. The top 10% of molecular structures (n = 20,000), as ranked by the Glide docking scores, were re-docked using two additional docking programs, eHiTs [19] and ICM [20]. A total of 22 molecules had consistent docking poses across the three programs: Glide, eHiTS, and ICM (RMSD ≤ 3A), and they were selected for in vitro testing.

### 2.2. Large-Scale In Silico Screening

Based on the hot spots identified from the in silico druggability assessment as described above, we performed a large-scale virtual screening of 7.6 million drug-like molecules from the ZINC15 database [17] (please see the Materials and Methods section for details on the binding site). Using our established virtual screening protocols [11,14], the binding poses and scores of these 7.6 million molecules were calculated using three docking programs—Glide [18], ICM [20], and OEDocking [21]. High-scoring small molecules with consistent docking poses (evaluated by the corresponding root-mean-square deviation, RMSD) were subjected to ADMET (absorption, distribution, metabolism, excretion, toxicity) and pharmacokinetics filtering using computational programs including ADMET Predictor [22], FAF-Drugs [23], and Quantitative Estimate of Drug-likeness (QED) [24]. Based on the consensus scoring of the above computational predictions (e.g., docking, RMSD, ADMET), we selected 118 top candidate compounds for experimental validation, including 66 compounds with molecular weights greater than or equal to 350 Dalton, and 52 compounds with molecular weights lower than 350 Dalton.

### 2.3. In Vitro Experimental Validation

A total of 140 compounds (including 22 compounds selected from the initial in silico screen and 118 compounds from the large-scale screen) were experimentally screened using TOX-dependent CTCL cells Hut78 cells at 10 µM and 100 µM concentrations. For the 18 compounds that showed concentration dependent inhibition of cell viability in Hut78 cells, their IC_50_ values were determined in 3 TOX-high/-dependent CTCL cell lines (Hut78, SZ4, Jurkat), and 3 TOX-low/-independent lymphoid cell lines (K562, U937, Mac2A) (Table 1 and Supplementary Table S1). These 18 compounds showed IC_50_ values that were lower in the TOX-high/-dependent cell lines than the TOX-low/-independent cell lines, as ranked by the TOX-selectivity index (Table 1). Several of these small molecule inhibitors (SMIs), such as 190444 and 190414, had IC_50_ values in the range of ~10–20 µM, more active than the hit compound 190010 that was identified from the initial in silico screen.

**Figure 3:**
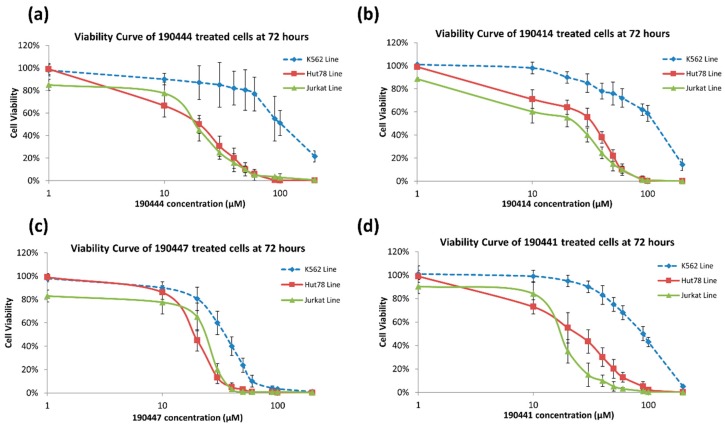
Viability curves of TOX-high and TOX-low expressing cells. Cells were treated with various concentrations of compounds, (**a**) 190444, (**b**) 190414, (**c**) 190447 and (**d**) 190441, for 72 h in a 37 °C incubator with 5% CO_2_. Viability was measured by CellTiter-Blue^®^ assay and compared to the DMSO control, as described in Materials and Methods. Jurkat and Hut78 cells (solid line) were the TOX-high-expressing cell lines, while K562 (dotted line) was the TOX-low-expressing cell line.

**Figure 4:**
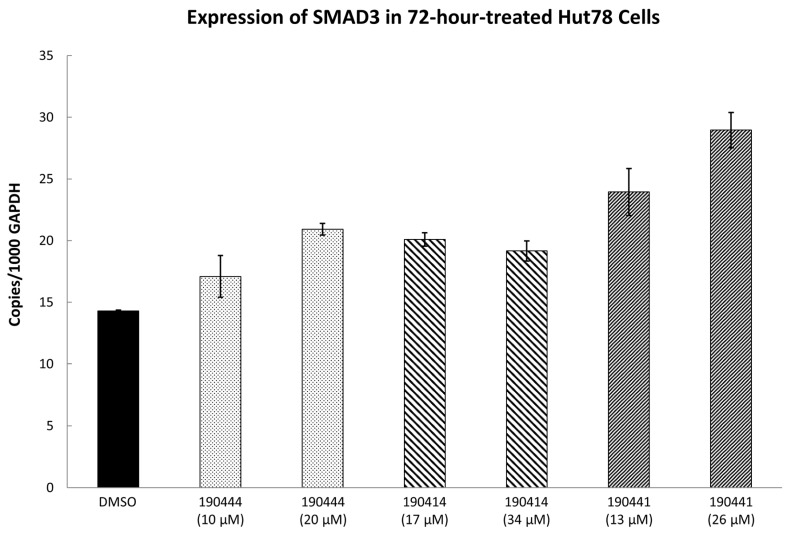
*SMAD3* expression in compound-treated Hut78 cells. Hut78 cells were treated with compounds 190444, 190414 and 190441 at their corresponding IC_50_ and half of IC_50_ concentrations for 72 h before RNA extraction for realtime measurement of the *SMAD3* expression level. Primers for *SMAD3* forward—CCC AGA GCA ATA TTC CAG AGA C, reverse—GTC CAT GCT GTG GTT CAT CT. Expression level of *SMAD3* was normalized by internal level of glyceraldehyde-3-phosphate dehydrogenase (GAPDH), as described in Materials and Methods.

As further illustrated in Figure 3, compounds 190444, 190414, 190447 and 190441 inhibited cell viability of the TOX-high cells (Hut78, Jurkat) selectively, compared to the TOX-low cells (K562). In addition, Figure 4 shows that compounds 190444, 190414 and 190441 increased the expression of SMAD3, which is normally suppressed by TOX [8].

## 3. Discussion

**Figure 5:**
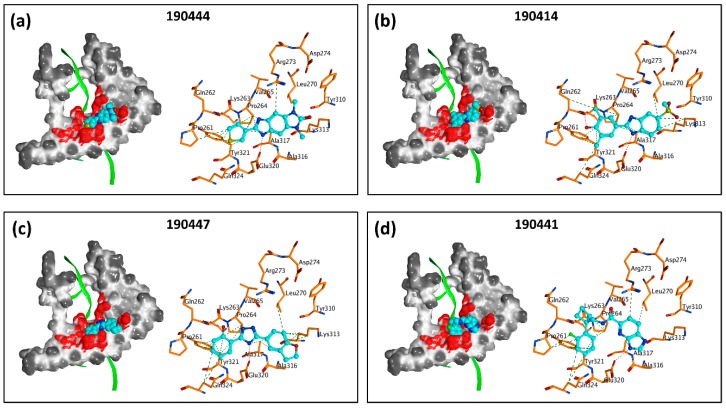
TOX small molecule inhibitors (SMIs) bind at the hot spots on the protein–DNA interface. Docking poses are shown for compounds (**a**) 190444, (**b**) 190414, (**c**) 190447, and (**d**) 190441 on the HMG-box domain of TOX (PDB: 2CO9). For each panel: (**left**) molecular surface representation, SMI in cyan, DNA in green ribbons, hot spots as red surface patches; (**right**) in-depth molecular interactions between SMI (cyan) and protein residues of TOX (orange), hydrogen bonds in red dotted lines, hydrophobic interactions in green dotted line. The PDB structure 2CO9 was used in docking. The DNA is shown here for illustration purpose (superimposed from 3TMM, as in Figure 1C), but not included in docking.

While previous studies [10] have established TOX as a promising drug target for CTCL therapies, there is a lack of small molecules that can directly inhibit TOX. By utilizing an established computer-aided drug design (CADD) platform [11] followed by in vitro experimental validation, we discovered a list of 18 small molecules that can inhibit the viability of TOX-high/-dependent cells with micromolar IC_50_ and up to 4-fold selectivity (Table 1). As illustrated in Figure 5, compounds 190444, 190414, 190447 and 190441 can bind at the hot spots located in close proximity to the protein–DNA interface on the HMG-box domain of TOX. These SMIs interact with TOX protein residues including Gln262, Pro264, Arg273, Lys313, Glu320, and Gln324 through hydrogen-bond and hydrophobic interactions, corresponding well to the hot spots as identified from the druggability assessment (Figure 2 and Figure 5). Most of the active molecules identified share common substructures including diazole, triazole and imidazolidinedione, the NH group of which forms a hydrogen bond with the residues such as Gln262 and Glu320. These common substructures could be utilized as a part of a pharmacophore model for selecting new compounds.

It is likely that such small molecule binding could interfere with TOX–DNA interactions and thus inhibit the transcriptional activity of TOX. This hypothesis is partially supported by the experimental results where compounds 190444, 190414 and 190441 increased the expression of SMAD3, which is normally suppressed by TOX (Figure 4). To establish the hypothesis further, additional experiments, including direct binding, DNA competition, and luciferase reporter assays are required. Future development of drug candidates inhibiting TOX–DNA interactions could follow previous studies where SMIs have been successfully developed via CADD to target the DNA-binding domains of other cancer drug targets, such as AR [12], ERG [14], and MYC [15].

Drug discovery is an expensive process, taking an average of 3 billion dollars and at least 10 years to bring a drug from laboratories to patients [25]. As demonstrated here, the use of virtual screening has significantly reduced the time and cost required during the drug discovery phase. By experimentally testing only 140 compounds, as selected from molecular docking of 7.6 million chemical structures, a total of 18 active hits were identified, thus achieving a hit rate of 13% (18/140), much higher than the hit rate (<1%) from conventional, experimental high-throughput screening without any computational guidance [11,26]. These 18 hit compounds provide a foundation on which more potent TOX-SMIs can be developed through 2D/3D similarity searches of chemical analogs against the entire ZINC database, which has grown exponentially from 700,000 compounds in 2005 [27] to over 1 billion molecules in 2019 [28]. This database of chemicals provides tremendous opportunity for TOX drug discovery, where virtual screening by molecular docking can be expanded from the initial 7.6 million to all of the 1 billion molecules. While molecular docking is already much faster than in vitro screening, docking 7.6 million small molecules against TOX still took 15 days (using 100 CPU cores). Applying the same docking algorithm (with the same amount of CPUs) on 1 billion chemical structures would take 2000 days or 5 years. We have previously developed a method of progressive docking that trains a machine learning model to efficiently predict binding scores based on chemical structures; thus, computer-intensive docking only needs to be performed on a subset of molecules pre-calculated as good target-binding candidates [29]. We anticipate the application of such a progressive docking algorithm (which would speed up screening approximately 50×) to virtually screen 1 billion molecules against TOX.

We also built a quantitative structure–activity relationship (QSAR) [30] model based on the 140 compounds which were experimentally tested. The overall accuracy of the model was about 92%. However, due to the small sample size and imbalance between the number of active vs. inactive compounds, the accuracy of the active class was 47% and the accuracy of the inactive class was 100%. While these accuracy values were better than random chance (active: 7% and inactive: 93%), the QSAR model needs to be further improved. Both of the approaches discussed above (similarity search and progressive docking) could greatly expand our current collection of TOX hit compounds and enable us to improve the QSAR model, which could guide future development for the next generation of potent and selective TOX drug candidates.

## 4. Materials and Methods

### 4.1. Structural Evaluation of TOX Druggability

The full-length protein sequence of the TOX protein (UniProt ID: O94900-1) was searched against the Protein Data Bank (PDB) [16] and a NMR structure of mouse TOX (PDB ID: 2CO9) was identified as the best matching template, with 100% sequence identity across the 87 amino acids of the HMG-box domain compared to the human TOX protein (from K251 to Y337, with residue numbering based on O94900-1).

To identify suitable sites for small molecule binding on the HMG-box domain of TOX, a total of 200,000 drug-like molecules (in-stock, 3D representation, with molecular weights from 375 to 400 Dalton, and logP = 1.5 +/−1) were extracted from the ZINC15 database [17]. The protein structure for the HMG-box domain of TOX (PDB ID: 2CO9) was then prepared using the Protein Preparation Wizard of Schrodinger 2016-3 software (with OPLS3 force field) [31]. A docking grid was set up to cover the entire TOX HMG-box domain, enabling ‘blind docking’ where no binding site was pre-defined. Each of the 200,000 small molecules was docked against the entire protein grid using the program Glide [18] (standard precision mode with default parameters) from the Schrodinger 2016-3 package (Schrödinger, New York, NY, USA). The top docking pose of each molecule was imported into the molecular operating environment (MOE, Chemical Computing Group, Montreal, Québec, CA) [32], and the Protein Ligand Interaction Fingerprints (PLIF) module was applied to all the 200,000 docking poses to calculate the frequency of interactions of each protein residue on the TOX HMG-box domain. Hot spots were determined as those protein residues with frequencies of interactions greater that or equal to 10%.

The 2CO9 structure from PDB was not in complex with DNA. Thus, to determine the TOX–DNA interface, 2CO9 was superimposed onto the HMG-box protein Transcription factor A, mitochondrial (TFAM) in complex with DNA (PDB ID: 3TMM) [33] using the protein alignment tool in the MOE [32].

### 4.2. In Silico Screening (Initial)

The top 10% or 20,000 virtual molecules (as ranked by the Glide docking scores [18]) were re-docked using another program, eHiTs [19] (default parameters). Similarly to the blind docking setup in Glide, no binding site on the TOX HMG-box domain was pre-defined in eHiTS. The top eHiTS pose was reported for each of the 20,000 molecules and compared to its corresponding Glide pose. It was identified that a total of 136 molecules demonstrated consistent docking poses with RMSD ≤ 3A between Glide and eHiTS. These 136 structures were re-docked again by the third program, ICM (Molsoft LLC, San Diego, CA, USA) [20] (default parameters), with a blind docking setup. Among the processed compounds, 22 demonstrated docking consistency with all three programs: Glide, eHiTS, and ICM. Through in vitro testing on TOX-expressing Sezary cell lines, one of the candidates, VPC-190010, showed inhibition activity with IC_50_ lower than 50 µM in all the TOX-expressing Sezary cell lines (data not shown).

### 4.3. In Silico Screening (Large-Scale)

The binding site for large-scale in silico screening was chosen based on the docking pose of the initial hit compound VPC-190010, which interacts with TOX hot spot residues including Gln262, Pro264, Arg273, Lys313, Glu320, and Gln324. The process of molecule screening was divided into two rounds for (1) oral and (2) topical modes of delivery. Out of the entire ZINC15 database [17], molecules were filtered based on the already known properties for oral and topical applications, as well as information obtained from the initial in silico screening.

The first round of large-scale in silico screening focused on an oral mode of delivery. The molecules with molecular weights greater than or equal to 350 Dalton and logP ≥ −1 were extracted from the ZINC15 database. These molecules were subjected to drug-like filtering criteria from FAF-Drugs4 [23,34], and the number of rings was set to be between 4 and 6. As a result, a total of about 3 million molecules were retained and then docked with Glide (standard precision mode with default parameters) [18]. Molecules with docking scores lower than −5 (lower is better) were further docked with the FRED program, OEDocking (OpenEye Scientific Software, Santa Fe, NM, USA) [21] (up to 500 conformers were generated for each molecule and were docked using FRED with default parameters), and the corresponding RMSD values were calculated for the top poses. All the molecules with a RMSD ≤ 3A were retained and were docked again using ICM [20]. For the poses predicted by ICM (default parameters), RMSD values were calculated against Glide and only the molecules with RMSD ≤ 3A were retained. Within this set, predicted docking pKi was calculated for each molecule using a custom MOE SVL script [32]. Other properties like ADMET (absorption, distribution, metabolism, excretion, toxicity) and pharmacokinetics predictions were also calculated using computational programs such as ADMET Predictor (Simulations Plus, Lancaster, CA, USA) [22], FAF-Drugs [23], and quantitative estimate of drug-likeness (QED) [24]. In the next step, a consensus scoring method was used, based on the criteria mentioned in Supplementary Table S2. Molecules with total consensus scores greater than or equal to 7 were retained and then clustered together to remove the similar compounds (70% similarity). Finally, a total of 66 compounds were chosen for experimental validation.

The next round focused on finding the molecules for topical mode of delivery. From the ZINC15 database, molecules with weights lower than 350 Dalton and logP ≥ −1 were extracted. This set of molecules was subjected to filters based on lead-like properties from FAF-Drugs4 [23,34], as well as filters specific to topical application where only the molecules with charge = 0 and 2 ≤ LogP ≤ 4 were retained [35,36]. Apart from the above-mentioned filters, molecules with chiral centers ≤ 1 (vendors usually sell the racemic mixture), 2 ≤ number of rings ≤ 4 (molecules with 1 ring are too simplistic), rotatable bonds ≤ 6, and 2 ≤ hydrogen bond acceptors ≤ 7 were retained. A total of 4.6 million molecules were retained after applying all the filters. These molecules were docked using the Glide program, standard precision with all other parameters default. Molecules with docking scores lower than −5 (lower is better) were further docked with the FRED program, OEDocking (up to 500 conformers were generated for each molecule and were docked using FRED with default parameters). RMSD values were calculated between the poses predicted by FRED and Glide. All the molecules with a RMSD ≤ 3A were retained and were docked using ICM (default parameters). For the poses predicted by ICM, RMSD values against Glide were calculated, and only the molecules with RMSD ≤ 3A were retained. Within this set, predicted docking pKi was calculated for each molecule using a custom MOE SVL script. Other properties like ADMET and pharmacokinetics predictions were also calculated by using computational programs such as ADMET Predictor [22], FAF-Drugs [23], and quantitative estimate of drug-likeness (QED) [24]. In the next step, a consensus scoring method was used based on the criteria mentioned in Supplementary Table S3. Molecules with total consensus scores greater than 5 were retained and then clustered together to remove similar compounds (70% similarity). Finally, a total of 52 compounds were chosen for experimental validation.

### 4.4. In Vitro Screening

All compounds were dissolved in DMSO as 50mM stock solution and diluted into treating concentration with growth medium (RPMI 1640 (Hyclone, GE) containing 10% FBS (Thermo Fisher Scientific, Waltham, MA, USA) and 1× anti-anti (Antibiotic-Antimycotic, Thermo Fisher Scientific, Waltham, MA, USA)).

Suspension cell lines Hut78, Jurkat, K562, and U937 were purchased from ATCC. SZ4 and Mac2A cell lines were generous gifts from Dr. Ivan Litvinov [37]. Cell were cultured in the growth medium and collected at logarithmic growth phase (about 5–10 × 10^5^ cells/mL). Cells were seeded into 96 well culture plates (Nunc, Thermo Fisher Scientific, Waltham, MA, USA) with 10^4^/well. Cells were cultured with various concentrations of the testing compounds in 0.2% DMSO or DMSO control only (as 0 µM) in growth medium for 64–68 h in an incubator containing 5% CO_2_ at 37 °C.

Viability assay was performed using CellTiter-Blue^®^ Cell Viability Assay (Promega, Madison, WI, USA), and fluorescent signal (579 Ex/584 Em) was recorded after 2 h and 4 h of incubation time using a Glomax Multi detection System (Promega, Madison, WI, USA).

All treatments were done in triplicate, and the final value was calculated as mean of the three datasets after subtracting medium only background. Net fluorescent signals at various concentrations were then compared to the DMSO only control and calculated as percentage of surviving population. IC_50_ values were determined using AAT Bioquest IC_50_ calculator (https://www.aatbio.com/tools/ic50-calculator/) with proportion of cells surviving at a range of concentrations of each drug present in the culture medium.

RNA expression of *TOX* was measured using the dye intercalated Realtime PCR method described previously [8]. RNA was extracted from cells using a RNAeasy purification kit (Qiagen, Hilden, Germany), and cDNA template was produced using SuperScript™ VILO™ cDNA Synthesis Kit (Invitrogen, Thermo Fisher Scientifict, Carlsbad, CA, USA). Gene expression levels were expressed as mRNA copies per 1000 glyceraldehyde-3-phosphate dehydrogenase (*GAPDH*) copies by standardizing to internal housekeeping gene GAPDH. The primers used for realtime measurement were as follows: *GAPDH* forward AAGATCATCAGCAATGCCTCC, *GAPDH* reverse TGGACTGTGGTCATGAGTCCTT; *TOX* forward GTGCAGAAATCCTCCCCCAC, *TOX* reverse TTTGTCCCTCTGCATGCCC.

## 5. Conclusions

In recent years, TOX has been characterized as a promising drug target in cutaneous T cell lymphoma. To address the lack of anti-TOX therapies, we engaged a computer-aided drug design pipeline to virtually screen 7.6 million compounds against the protein–DNA interface on the HMG-box domain of TOX. As a result of the computational screen, a total of 140 compounds were selected for experimental validation, with 18 of them demonstrating sufficient inhibition of viability of TOX-high/-dependent CTCL cells with micromolar potency and up to 4-fold selectivity over TOX-independent cells. The success of the current stage highlights the need to screen the remaining (approximately 1 billion) drug-like compounds in the ZINC databases for the identification of additional molecules with anti-TOX activities. The compounds identified in this study will serve as prototypical TOX-inhibitors for developing the next generation of more potent and selective CTCL therapeutics through further chemical optimization.

## Figures and Tables

**Table 1:** Top candidates for TOX small molecule inhibitors (SMIs).

Compound VPC-ID	Chemical Structure ^1^	Average IC_50_ (µM) (TOX-High Cells) ^2^	Average IC_50_ (µM) (TOX-Low Cells) ^3^	TOX-Selectivity Index ^4^
190444	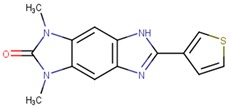	16.28 ± 1.64	69.94 ± 14.66	4.30
190414	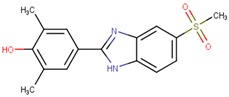	20.64 ± 5.59	69.55 ± 20.62	3.37
190350	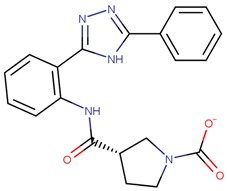	12.01 ± 2.65	33.69 ± 7.15	2.80
190447	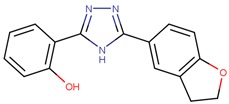	16.68 ± 3.32	41.13 ± 3.59	2.47
190410	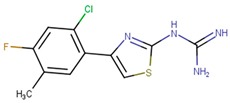	15.35 ± 1.77	37.45 ± 5.13	2.44
190358	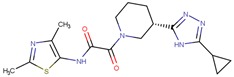	15.13 ± 4.34	32.98 ± 5.35	2.18
190441	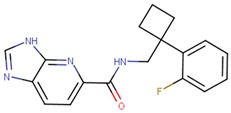	34.76 ± 12.67	64.69 ± 13.83	1.86
190327	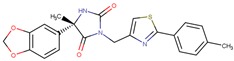	16.55 ± 1.96	30.29 ± 8.09	1.83
190343	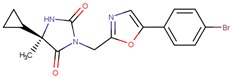	33.26 ± 5.57	56.29 ± 5.09	1.69
190341	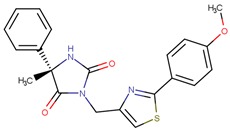	52.44 ± 3.96	85.82 ± 8.36	1.64
190325	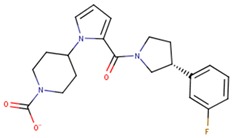	39.77 ± 6.20	64.63 ± 7.39	1.63
190323	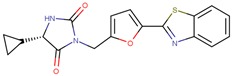	43.79 ± 4.94	68.68 ± 9.51	1.57
190339	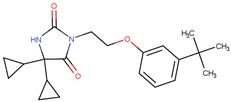	34.02 ± 4.14	51.83 ± 3.19	1.52
190322	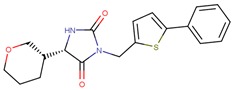	24.64 ± 4.74	36.19 ± 7.14	1.47
190349	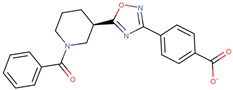	52.88 ± 11.76	70.57 ± 10.05	1.33
190301	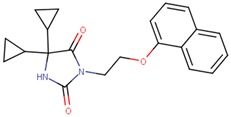	38.75 ± 1.94	47.29 ± 3.97	1.22
190354	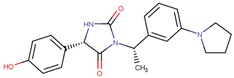	58.59 ± 3.80	66.12 ± 11.85	1.13
190010	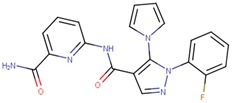	37.70 ± 8.11	n/a ^5^	n/a ^5^

^1^ The chemical isomer from the docking model is shown here, while a racemic mixture was used in the in vitro experiments due to vendor availability. ^2^ Average IC_50_ values of cell viability from three TOX-high/-dependent CTCL cell lines (Hut78, SZ4, Jurkat). ^3^ Average IC_50_ values of cell viability from 3 TOX-low/-independent lymphoid cell lines (K562, U937, Mac2A). ^4^ TOX-selectivity index = Average IC_50_ (TOX-Low cells)/Average IC_50_ (TOX-High cells). ^5^ Data not available.

## Supplementary Materials

The following are available online at https://www.mdpi.com/1420-3049/24/19/3459/s1, Table S1: IC_50_ summary from cell viability assays for the 18 TOX small molecule inhibitors, Table S2: Consensus Matrix for molecules with MW ≥ 350 Dalton (oral application), Table S3: Consensus Matrix for molecules with MW < 350 Dalton (topical application), PDB files for each of the 18 small molecules docked on the HMG-box domain of TOX (PDB: 2CO9).
