# Supplementary material for: Computer-Aided Discovery of Small Molecule Inhibitors of Thymocyte Selection-Associated High Mobility Group Box Protein (TOX) as Potential Therapeutics for Cutaneous T-Cell Lymphomas
Source: Molecules. 2019 Sep 24;24(19):3459. doi: 10.3390/molecules24193459 (PMC6803922; doi:10.3390/molecules24193459)
Supplement: Supplementary file 1 [file molecules-24-03459-s001.zip › Supplementry_Tables_S2_S3.docx]

**Table S2.** Consensus scoring matrix for molecules with MW ≥ 350 Dalton (oral application). The total score for each molecule is calculated based on the criteria mentioned in the table. Molecules with total consensus scores ≥ 7 are retained and are subjected to clustering.

| **Property** | **Value** | **Consensus Score** |
| --- | --- | --- |
| Glide Docking Score  (lower is better) | < -5.6 (top 10%) | 1 |
| Glide Efficiency  (lower is better) | < -0.21 (top 10%) | 1 |
| PAINS | 0 (No Problem) | 1 |
|  | 1 (Problem) | 0 |
| FAF-Drugs Result | 0 (Accepted) | 1 |
|  | 1 (Intermed) | 0.5 |
|  | 2 (Rejected) | -1 |
| Dock pK_i_  (higher is better) | ≥ 5 | 1 |
|  | ≥ 4 & < 5 | 0.5 |
|  | < 4 | 0 |
| RMSD  (lower is better) | ≤ 1 | 1 |
|  | ≤ 2 & > 1 | 0.5 |
|  | > 2 | 0 |
| ADMET Risk  (lower is better) | ≤ 1 | 1 |
|  | ≤ 2 & > 1 | 0.5 |
|  | > 2 | 0 |
| QED_w_ Score  (higher is better) | > 0.5 | 1 |
|  | ≤ 0.5 | 0 |

**Table S3.** Consensus scoring matrix for molecules with MW<350 Dalton (topical application). The total score for each molecule is calculated based on the criteria mentioned in the table. Molecules with total consensus scores > 5 are retained and are subjected to clustering.

| **Property** | **Value** | **Consensus Score** |
| --- | --- | --- |
| Glide Docking Score  (lower is better) | < -5.51 (top 10%) | 1 |
| Glide Efficiency  (lower is better) | < -0.27 (top 10%) | 1 |
| FAF-Drugs Result | 0 (Accepted) | 1 |
|  | 1 (Intermed) | 0.5 |
|  | 2 (Rejected) | -1 |
| Dock pKi  (higher is better) | ≥ 4.65 (top 10%) | 1 |
| RMSD  (lower is better) | ≤ 1 | 1 |
|  | ≤ 2 & > 1 | 0.5 |
|  | > 2 | -0.5 |
| ADMET Risk  (lower is better) | ≤ 1 | 1 |
|  | > 1 | 0 |
| QED_w_ Score  (higher is better) | > 0.9 (top 10%) | 1 |
